# Gut microbiota profiles in two New Zealand cohorts with overweight and prediabetes: a Tū Ora/PREVIEW comparative study

**DOI:** 10.3389/fmicb.2023.1244179

**Published:** 2023-11-16

**Authors:** Akarsh Mathrani, Louise W. Lu, Ivana R. Sequeira-Bisson, Marta P. Silvestre, Michael Hoggard, Daniel Barnett, Mikael Fogelholm, Anne Raben, Sally D. Poppitt, Michael W. Taylor

**Affiliations:** ^1^School of Biological Sciences, University of Auckland, Auckland, New Zealand; ^2^High-Value Nutrition National Science Challenge, Auckland, New Zealand; ^3^Human Nutrition Unit, University of Auckland, Auckland, New Zealand; ^4^Centro de Investigação em Tecnologias e Serviços de Saúde (CINTESIS), NOVA University of Lisbon, Lisbon, Portugal; ^5^Department of Statistics, University of Auckland, Auckland, New Zealand; ^6^Department of Food and Nutrition, Faculty of Agriculture and Forestry, University of Helsinki, Helsinki, Finland; ^7^Department of Nutrition, Exercise and Sports, Faculty of Science, University of Copenhagen, Copenhagen, Denmark; ^8^Clinical Research, Copenhagen University Hospital – Steno Diabetes Center Copenhagen, Herlev, Denmark; ^9^Department of Medicine, University of Auckland, Auckland, New Zealand

**Keywords:** bacterial diversity, prediabetes, ethnicity, *Firmicutes*, *Bacteroidetes*

## Abstract

Obesity-related metabolic diseases such as type 2 diabetes (T2D) are major global health issues, affecting hundreds of millions of people worldwide. The underlying factors are both diverse and complex, incorporating biological as well as cultural considerations. A role for ethnicity – a measure of self-perceived cultural affiliation which encompasses diet, lifestyle and genetic components – in susceptibility to metabolic diseases such as T2D is well established. For example, Asian populations may be disproportionally affected by the adverse ‘TOFI’ (Thin on the Outside, Fat on the Inside) profile, whereby outwardly lean individuals have increased susceptibility due to excess visceral and ectopic organ fat deposition. A potential link between the gut microbiota and metabolic disease has more recently come under consideration, yet our understanding of the interplay between ethnicity, the microbiota and T2D remains incomplete. We present here a 16S rRNA gene-based comparison of the fecal microbiota of European-ancestry and Chinese-ancestry cohorts with overweight and prediabetes, residing in New Zealand. The cohorts were matched for mean fasting plasma glucose (FPG: mean ± SD, European-ancestry: 6.1 ± 0.4; Chinese-ancestry: 6.0 ± 0.4 mmol/L), a consequence of which was a significantly higher mean body mass index in the European group (BMI: European-ancestry: 37.4 ± 6.8; Chinese-ancestry: 27.7 ± 4.0 kg/m^2^; *p* < 0.001). Our findings reveal significant microbiota differences between the two ethnicities, though we cannot determine the underpinning factors. In both cohorts *Firmicutes* was by far the dominant bacterial phylum (European-ancestry: 93.4 ± 5.5%; Chinese-ancestry: 79.6 ± 10.4% of 16S rRNA gene sequences), with *Bacteroidetes* and *Actinobacteria* the next most abundant. Among the more abundant (≥1% overall relative sequence abundance) genus-level taxa, four zero-radius operational taxonomic units (zOTUs) were significantly higher in the European-ancestry cohort, namely members of the *Subdoligranulum*, *Blautia*, *Ruminoclostridium,* and *Dorea* genera. Differential abundance analysis further identified a number of additional zOTUs to be disproportionately overrepresented across the two ethnicities, with the majority of taxa exhibiting a higher abundance in the Chinese-ancestry cohort. Our findings underscore a potential influence of ethnicity on gut microbiota composition in the context of individuals with overweight and prediabetes.

## 1. Introduction

Type 2 diabetes (T2D) is a significant global health issue and among the fastest growing chronic disease worldwide (International Diabetes Federation, 2021). When considering prediabetes, where individuals develop dysglycaemia indicative of increased risk of later T2D, the situation is even starker. Among the ~5.5 million residents of New Zealand, for example, it is estimated that ~4% have T2D (Te Whatu Ora: Health New Zealand, 2021) and a further ~20% have prediabetes (New Zealand Society for the Study of Diabetes, 2023). Fortunately, there is strong evidence that lifestyle (including dietary) change in those with prediabetes can delay or even prevent progression to frank T2D (Liu et al., 2015). It is therefore of considerable importance to understand the factors which influence prediabetes. Ethnicity, defined here as a measure of self-perceived cultural affiliation, encompassing diet, lifestyle as well as underlying genetics, is one such factor. An association between ethnicity and susceptibility to obesity, T2D and other metabolic disorders is well documented (Spanakis and Golden, 2013; Yip et al., 2017). Certainly, differences in glucose metabolism and insulin resistance have been observed between ethnic groups, but with complex causes (Golden et al., 2012). Another potential contributor to (pre)diabetes is the gut microbiota (Deschasaux et al., 2018). Although the underlying mechanisms remain unclear, a combination of biological, clinical and social factors likely play a role, with metabolic susceptibility and microbiota profile also potentially associated through mechanisms yet to be determined.

Given that T2D progression is a result of both environmental and genetic factors, a higher T2D risk profile observed in Asian ethnicities at low body mass index (BMI) (Haldar et al., 2015) has been suggested to reflect a general inability to adapt to the Western lifestyle and dietary changes popularized with the rise of urbanization and increased globalization over the past two centuries (Akiyama, 2008). As the gut microbiota plays a prominent role in regulating host metabolic homeostasis (Sears, 2005; LeBlanc et al., 2013; Quigley, 2013; Clarke et al., 2014; Duvallet et al., 2017), the utility of microbial community characteristics as non-invasive diagnostic indicators of health has in recent years become widely recognized (Duvallet et al., 2017). However, although associations between disease pathogenicity and the gut microbiota have been established, the taxonomic composition is highly personalized among individuals (Gaulke and Sharpton, 2018) and the noise projected strongly influences the accuracy of microbiota-based investigations. Yet the extent of contribution, if any, to this inter-individual variation by ethnicity remains largely unresolved: indeed, large observational studies examining the gut microbiota of participants of varied ethnic backgrounds but with shared geography have produced findings both in support of ethnicity explaining more of the variation than environmental factors (in 2,084 participants) (Deschasaux et al., 2018), and vice versa (in 1,046 participants) (Rothschild et al., 2018).

The higher T2D risk profile in the absence of high BMI in Asian cohorts is characterized by adverse body fat distribution, including excess abdominal, visceral and ectopic fat deposition compared with, for example, European-ancestry individuals (Lear et al., 2007; Nazare et al., 2012; Jo and Mainous, 2018). An inability to expand subcutaneous fat stores (Misra and Khurana, 2011; Ramachandran et al., 2012) may contribute to this adverse Asian TOFI (Thin on the Outside, Fat on the Inside) phenotype (Thomas et al., 2012), where ectopic lipid infiltration into pancreas and liver (Dickinson et al., 2002; Liew et al., 2003; Cortés and Fernández-Galilea, 2015) may occur even in individuals with low BMI and whole body adiposity (WHO Expert Consultation, 2004). This condition may both inhibit glucose-mediated β-cell insulin secretion (Lee et al., 1994) and decrease insulin sensitivity (Shibata et al., 2007), and could potentially be associated with disparate microbiota.

The primary aim of this study was to describe the fecal microbiota in individuals with prediabetes. Previous research has implicated metabolic characteristics such as BMI and body weight as being of relevance, with ethnicity also potentially playing a role. We thus leveraged a subset of a previously published 16S rRNA gene dataset which focused on adults of European descent living in New Zealand [from the multi-national PREVIEW lifestyle intervention (Raben et al., 2021; Jian et al., 2022)], and an unpublished dataset from a cohort of Chinese-descent adults also residing in New Zealand (Tū Ora dietary intervention). Mean fasting plasma glucose (FPG) was matched between cohorts in this sub-analysis, a consequence of which was a higher mean BMI in the European group. The issue of confounding due to difference in BMI was mitigated through the use of permutational analysis of variance (PERMANOVA). Cohorts were defined and named in accordance with Statistics New Zealand terminology. This allowed us to consider differences which may relate to ethnicity as well as glucoregulatory markers and body composition.

## 2. Materials and methods

### 2.1. Study design and participants

This study utilized cross-sectional baseline data from two separate clinical trials, each focusing on prevention of T2D in individuals with overweight and prediabetes. The dataset from the Chinese-ancestry cohort was obtained from the Tū Ora dietary intervention, and the European-ancestry cohort from the PREVIEW lifestyle intervention (Raben et al., 2021; Jian et al., 2022). Cohorts were defined and named in accordance with Statistics New Zealand terminology. Both clinical trials were conducted at the University of Auckland Human Nutrition Unit, New Zealand, with study participants resident predominantly in Auckland (~85%), or in Wellington (~15%), New Zealand. Participants for the current analysis were selected from their respective larger cohorts based on confirmed prediabetes, defined by raised FPG of 5.6–6.9 mmol/L (American Diabetes Association, 2018) measured in clinic. The two cohorts were matched for mean FPG. Both cohorts were also required to meet the following inclusion criteria, as named in accordance with the original publications: self-reported ‘Chinese’ (Tū Ora) or ‘Caucasian’ (PREVIEW) descent, currently resident in New Zealand, 25–70 years of age, overweight or obese defined as BMI ≥23 kg/m^2^ for Chinese-ancestry adults and ≥25 kg/m^2^ for European-ancestry adults, Finnish Diabetes Risk Score (FINDRISC) ≥9 for Chinese-ancestry adults and ≥12 for European-ancestry adults indicative of increased future risk of T2D (Lindström and Tuomilehto, 2003; Silvestre et al., 2017), and stable body weight for 2 months prior to the trial (<5% change, self-reported). Exclusion criteria for both cohorts were: smoking, pregnancy, diagnosis of any significant co-morbidities such as diabetes or cancer, medications or supplements that may influence glycaemia (self-reported). None of the participants from either cohort reported antibiotic use within 1 month prior to the trial. All participants who met all inclusion, but none of the exclusion, criteria were selected from each cohort for this current study. Human ethics approval was granted by the Health and Disabilities Ethics Committee (HDEC), New Zealand, and participants provided written, informed consent before enrolment. Tū Ora was registered with the Australian New Zealand Clinical Trial Registry (Trial ID: ACTRN12618000476235) and PREVIEW with ClinicalTrials.gov (Trial ID: NCT01777893). Thirty-two Chinese-ancestry and 39 European-ancestry participants with overweight and prediabetes were selected for the purpose of this analysis.

### 2.2. Clinical assessments

Participants attended their clinic visit in the morning, following an overnight fast, with procedures conducted as previously described for the PREVIEW study (Fogelholm et al., 2017). Body weight, height, waist and hip circumference, and body composition were all measured in the clinic. Body weight was measured using digital scales (Mettler Toledo Spider 2, Zurich, Switzerland) and height using a wall-mounted stadiometer (Seca 222, Hamburg, Germany), both while lightly clad and without shoes. Waist and hip circumference were measured using a non-stretch tape (Abbott Laboratories, Illinois, USA). Waist was measured at the midpoint of the lowest palpable rib and the hip joint bone, around the belly and not under, and hip at the widest point over the greater trochanter. Body composition was assessed using dual energy x-ray absorptiometry (iDXA, GE Healthcare, Madison, WI, USA). Total body fat % and abdominal fat % estimates were calculated according to the formulae:


$$\mathrm{Total}\;\mathrm{body}\;\mathrm{fat}\;\left(\%\right)=\frac{\mathrm{total}\;\mathrm{body}\;\mathrm{fat}\;\mathrm{mass}\;\left(g\right)}{\begin{array}{c} \mathrm{total}\;\mathrm{body}\;\mathrm{fat}\;\mathrm{mass}\;\left(g\right)\\ +\mathrm{total}\;\mathrm{body}\;\mathrm{lean}\;\mathrm{mass}\;\left(g\right) \end{array}}\times 100$$

$$\mathrm{Abdominal}\;\mathrm{fat}\;\left(\%\right)=\frac{\mathrm{abdominal}\;\mathrm{fat}\;\mathrm{mass}\;\left(g\right)}{\begin{array}{c} \mathrm{abdominal}\;\mathrm{fat}\;\mathrm{mass}\;\left(g\right)\\ +\mathrm{abdominal}\;\mathrm{lean}\;\mathrm{mass}\;\left(g\right) \end{array}}\times 100$$

A fasted venous blood sample was also collected for analysis of glucose (FPG) and insulin. Demographic information for participants is presented in Table 1.

**Table 1:** Baseline characteristics of study participants.

	**European-ancestry cohort (*n* = 39)**	***n***	**Chinese-ancestry cohort (*n* = 32)**	***n***	***p*-val**	
**Demographic**						
Clinic site (Auckland: Wellington)	39:0	39	23:9	32	<0.001	*
Sex (male: female)	11:28	39	21:11	32	0.002	*
Age (years)	49.7 (46; 28–70)	39	48.1 (49; 28–72)	32	0.561	
**Anthropometry**						
Body weight (kg)	104.0 (107.1; 70–152)	39	77.6 (74; 59–120)	32	<0.001	*
Height (m)	1.7 (1.7; 1.5–1.8)	39	1.7 (1.7; 1.5–1.8)	32	0.992	
Body mass index, BMI (kg/m^2^)	37.4 (36.1; 28–63)	39	27.7 (27.6; 23–40)	32	<0.001	*
Waist circumference (cm)	111 (112; 82–143)	39	95.6 (92.9; 77–130)	32	<0.001	*
Hip circumference (cm)	122.1 (119; 101–163)	39	103.2 (101.5; 91–130)	32	<0.001	*
Waist: Hip ratio	0.9 (0.9; 0.8–1.1)	39	0.9 (0.9; 0.8–1.1)	32	0.439	
**Body composition**						
Total body fat (%)	44.9 (46.2; 25–57)	29	31.6 (31.4; 22–43)	21	<0.001	*
Abdominal fat (% of total abdominal mass)	52.4 (53.7; 35–64)	29	40.3 (40.1; 30–53)	21	<0.001	*
**Glucoregulatory markers**						
Fasting plasma glucose, FPG (mmol/L)	6.1 (6; 5.6–6.9)	39	6.0 (5.8; 5.6–6.8)	32	0.221	
Fasting insulin (μU/mL)	12.0 (9.7; 4.0–31.8)	29	16.7 (15.1; 1.8–44.0)	32	0.04	*
HOMA-IR index	3.3 (2.5; 1.1–8.8)	29	4.4 (3.9; 0.5–11.1)	32	0.05	*

Mean (median; range). Missing fasting insulin data in the European-ancestry cohort are due to limitations in available metadata; missing body composition data for either cohort are due to no DXA scan. *Value of *p*: Fisher test was utilized for categorical variables clinic site and sex, with unpaired two sample *t*-tests used for all continuous variables.

### 2.3. Blood analyses

FPG was measured using the hexokinase method (Cobas C311 analyzer, Roche Diagnostics, Indianapolis, USA) from plasma samples in Chinese-ancestry participants, and whole blood with manual conversion to plasma glucose in European-ancestry participants (Reflotron Plus Desk Top Analyser, Mannheim, Germany) (Kim, 2016). Insulin was analyzed using Elecsys immunoassay with electrochemiluminescence technology (Cobas e411 Analyser, Roche Diagnostics, Indianapolis, USA).

### 2.4. Fecal sample collection

Fecal sample collection, processing and microbiological analyses were performed for both studies in the same laboratory using identical methodologies. Fecal samples were collected at home by participants into a sterile collection tube, frozen at −18°C in their home freezers and then delivered to the research clinic in a chilly bin without sample thawing (sample tubes were encased in an “ice jacket” to prevent rapid thawing). All fecal samples were then stored in the clinic at −80°C prior to DNA extraction.

### 2.5. DNA extraction

Genomic DNA was extracted from 0.25 g fecal sample using International Human Microbiome Standards (IHMS) Protocol #9 (Costea et al., 2017), which is a repeated bead-beating method utilizing 0.1 mm silica and 3 mm glass beads. Cell lysis was performed using a non-commercial lysis buffer recipe (500 mM NaCl, 50 mM Tris–HCl at pH 8.0, 50 mM EDTA and 4% SDS) as per the protocol, however a Qiagen Tissuelyser II (Retch) was used (frequency of 30 Hz, for two cycles of 1.5 min) to break cells instead of the FastPrep® -24 Instrument advised by the protocol. A QIAamp DNA Minikit (Qiagen, 51306) was utilized in the final steps of the protocol for removal of RNA, protein and for DNA purification, as recommended by the protocol. Negative control extractions containing 250 μL of sterile water instead of 0.25 g fecal sample were also carried out to test for potential contamination. All extracts were subsequently analyzed on a Nanodrop 3,300 fluorospectrometer (Nanodrop Technologies Inc., Wilmington, USA) to determine DNA quality and concentration.

### 2.6. Bacterial 16S rRNA gene amplicon sequencing

Bacterial community structure was analyzed by polymerase chain reaction (PCR) amplification, then sequencing of the highly variable V3-V4 region of the 16S rRNA gene. The KAPA High Fidelity HotStart Readymix PCR Kit (Kapa Biosystems®) was utilized, with ~50 ng of template genomic DNA used per reaction. Labeled with Illumina MiSeq-compatible adaptors, the widely used primer pair 341F (5’-CCTACGGGNGGCWGCAG-3’) and 785R (5’-GACTACHVGGGTATCTAATCC-3’) (Klindworth et al., 2013) was used with the following thermocycling conditions: initial denaturation and activation of enzymes at 95°C for 3 min, followed by 25 cycles of denaturation (95°C for 30 s), annealing (55°C for 30 s) and elongation (72°C for 30 s), with a final extension of 72°C for 10 min. PCR products were electrophoresed on 1% (w/v) agarose gels with SYBR Safe nucleic acid stain (Invitrogen Co., USA) to ensure correct amplicon size. Negative PCR controls, in which nuclease-free H_2_O was used instead of template DNA, as well as amplifications of eluates from the negative DNA extractions, did not produce any visible products. Randomly selected negative controls were nonetheless submitted for sequencing even if no product was visible on an agarose gel. PCR amplicons were purified using AMPure magnetic beads (Beckman-Coulter Inc., USA) in accordance with the manufacturer’s instructions, and quantified using a Qubit dsDNA high-sensitivity kit (Invitrogen Co., USA). Standardized concentrations of the purified samples were submitted to Auckland Genomics Ltd. for Illumina MiSeq sequencing (2 × 300 bp chemistry).

### 2.7. Bioinformatics

A bioinformatics pipeline previously described by Hoggard et al. (2019), and compatible with the USEARCH (Edgar, 2010) software package, was utilized to join the raw pairs of 16S rRNA gene sequence reads, remove primer-binding regions, quality filter and study the merged sequences (Edgar et al., 2011). Merged sequence reads were further error-corrected and zero-radius operational taxonomic units (zOTUs) of 100% similarity were generated (Edgar, 2016a). Non-target (e.g., human-derived) sequences were removed and the SILVA v123 database used to assign taxonomy to each zOTU (Quast et al., 2013; Edgar, 2016b). All unassigned sequences were manually checked via BLAST nucleotide search and any sequences producing non-target (i.e., not bacteria) hits were removed (Johnson et al., 2008). The sequence depth of each sample was rarefied to 2,405 reads (range: 3–69,963), which was the lowest practical number of reads obtained across samples, to ensure an even number of sequences would be compared for each sample during downstream statistical analysis. The 16S rRNA gene sequence data from the Chinese-ancestry cohort were deposited in Genbank under SRA Bioproject PRJNA900794, and European-ancestry cohort data in the European Nucleotide Archive under accession number PRJEB43667.

### 2.8. Statistical analysis

The statistical environment R was used for all analyses (R Core Team, 2022), with a value of *p* <0.05 considered to be statistically significant. Statistical analysis was performed using all available data from all participants. Fisher test was utilized for testing participant distribution by categorical parameters (i.e., clinical site and sex), while distribution by all other continuous constraints was tested with a two-sample *t*-test. A two-sample *t*-test was also used to compare gut microbiota diversity between cohorts and Bray–Curtis dissimilarity was utilized for the non-metric multidimensional scaling (nMDS) analysis of bacterial community structure. Significance of variance was determined with PERMANOVA, performing 9,999 unrestricted permutations of the raw data. Covariate analyses with PERMANOVA included testing for associations between ethnicity and key demographic variables (i.e., age and sex) and metabolic variables (i.e., FPG and BMI). Further details pertaining to the PERMANOVA analysis, including the R script used, are provided in the Supplementary material. Differential zOTU abundances were calculated using the log2 fold-change of counts data (based on European-ancestry cohort as the reference factor, representing a value of 0). Standard error (SEM) estimates were used for the log2 fold-change values and the Wald test was performed for the generation of *p*-values (subsequently adjusted for false discovery rate using the Benjamini–Hochberg procedure).

## 3. Results

The European-ancestry and Chinese-ancestry cohorts both demonstrated dysglycaemia characteristic of prediabetes (mean ± SD: European-ancestry 6.1 ± 0.4; Chinese-ancestry 6.0 ± 0.4 mmol/L). They were also of similar age and height but with differences in mean body weight (104.0 ± 17.4; 77.6 ± 14.2 kg), BMI (37.4 ± 6.8; 27.7 ± 4.0 kg/m^2^), waist and hip circumference, total body fat (44.9 ± 8; 31.6 ± 6%) and abdominal fat (52.4 ± 8.7, 40.3 ± 6.7%), all of which were significantly lower in Chinese-ancestry participants (*p* < 0.001). Whilst the two cohorts had similar FPG, fasting insulin was significantly higher in Chinese-ancestry participants (European-ancestry 12 ± 6.9 μU/mL; Chinese-ancestry 16.7 ± 9.9 μU/mL; *p* = 0.04). Sex distribution also differed between ethnicities (European-ancestry 11 M:28F; Chinese-ancestry 21 M:11F; *p* = 0.002). Demographic, anthropometric, metabolic and body composition data for the two cohorts are presented in Table 1.

**Figure 1:**
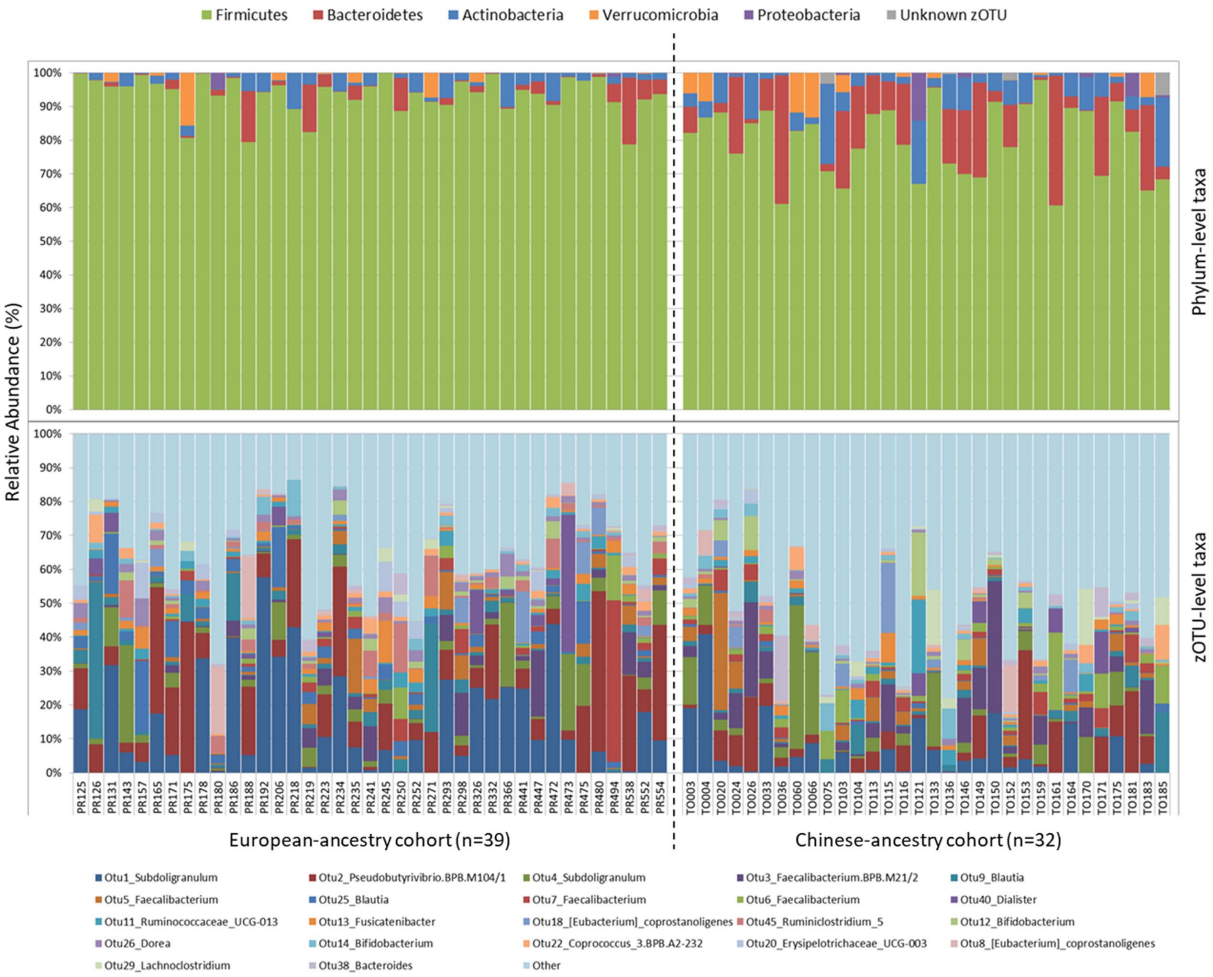
Phylum-level **(top)** and zOTU-level **(bottom)** gut bacterial community composition for European-ancestry and Chinese-ancestry cohorts. zOTUs with ≥1% overall 16S rRNA gene relative sequence abundance are shown, with all remaining zOTUs grouped together in “Other”.

In both cohorts, *Firmicutes* was by far the most abundant bacterial phylum (Figure 1), though its average relative sequence abundance was significantly higher among European-ancestry individuals (mean ± SD: 93.4 ± 5.5%) than Chinese-ancestry individuals (79.6 ± 10.4%; *p* < 0.001). The bacterial phyla *Bacteroidetes* (*p* = 0.001) and *Actinobacteria* (*p* = 0.006) also differed significantly between the cohorts, with both occurring at similar relative abundance in European-ancestry participants (*Bacteroidetes =* 2.7 ± 4.5%; *Actinobacteria* = 2.7 ± 2.7%), but *Bacteroidetes* (11 ± 11.2%) being more abundant than *Actinobacteria* (6.2 ± 5.8%) in Chinese-ancestry participants (*p >* 0.05). Members of the phyla *Verrucomicrobia* and *Proteobacteria* were also present in both cohorts, though their respective populations did not differ significantly between the cohorts. At a finer taxonomic level (i.e., zOTU), there was considerable inter-individual variation, however among those zOTUs with ≥1% overall relative sequence abundance, four taxa were significantly more abundant in European-ancestry participants, namely *OTU1_Subdoligranulum* (*p* = 0.01), *OTU25_Blautia* (*p* = 0.002), *OTU45_Ruminoclostridium_5* (*p* = 0.004) and *OTU26_Dorea* (*p* = 0.007).

**Figure 2:**
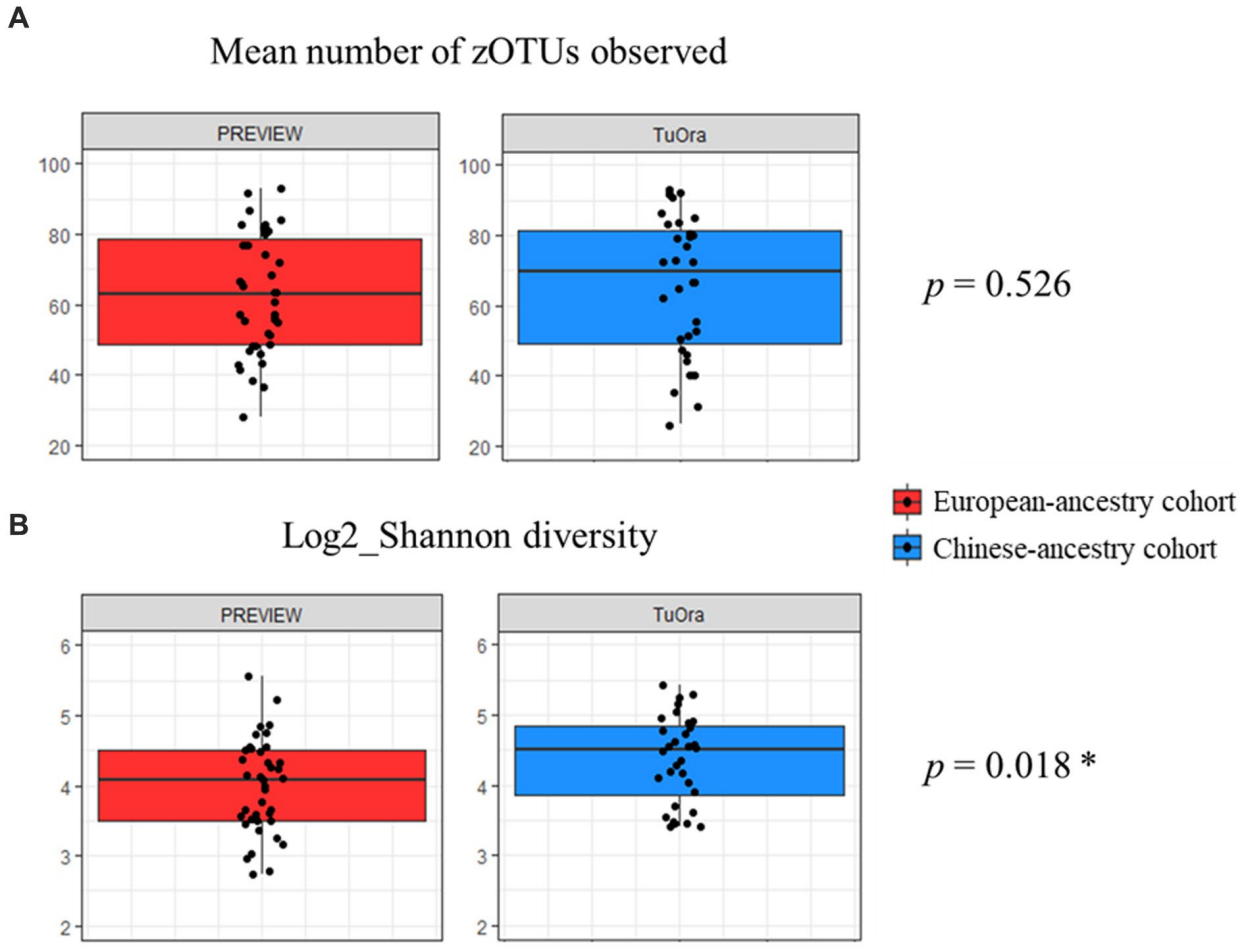
Mean alpha diversity parameters compared between the two ethnicity cohorts. *P*-values determined using unpaired *t*-test. **(A)** Mean number of observed zOTUs for each cohort. **(B)** Mean log2_Shannon diversity index of observed zOTUs for each cohort.

**Figure 3:**
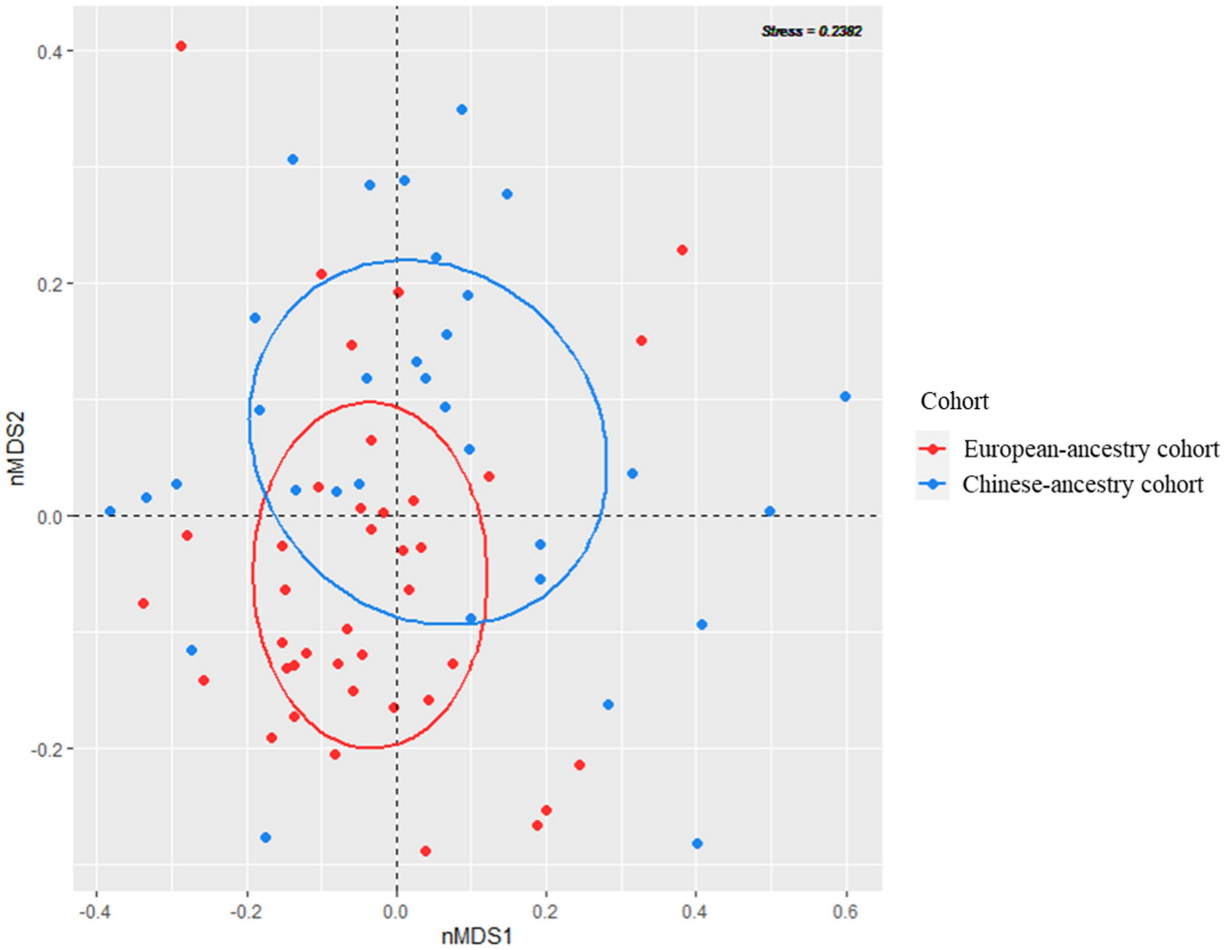
Visualization of bacterial community beta diversity in the two ethnicity cohorts, using non-metric multidimensional scaling (nMDS) based on Bray–Curtis dissimilarity.

Concerning alpha diversity (Figure 2), bacterial richness (number of observed zOTUs) did not differ significantly between the two cohorts (*p* = 0.526), but zOTUs were more evenly distributed in the Chinese-ancestry cohort (Shannon diversity *p* = 0.018). PERMANOVA revealed a highly significant difference in bacterial community composition between the two cohorts (*p* < 0.001; r^2^ = 0.051), with the outcome that ethnicity but not age, sex, BMI or FPG was identified as being significantly associated with microbiota composition when these key factors were tested against each other. This ethnicity-correlated difference in beta diversity was evident in the observed clustering of samples (albeit with some overlap) in the nMDS plot (Figure 3).

**Figure 4:**
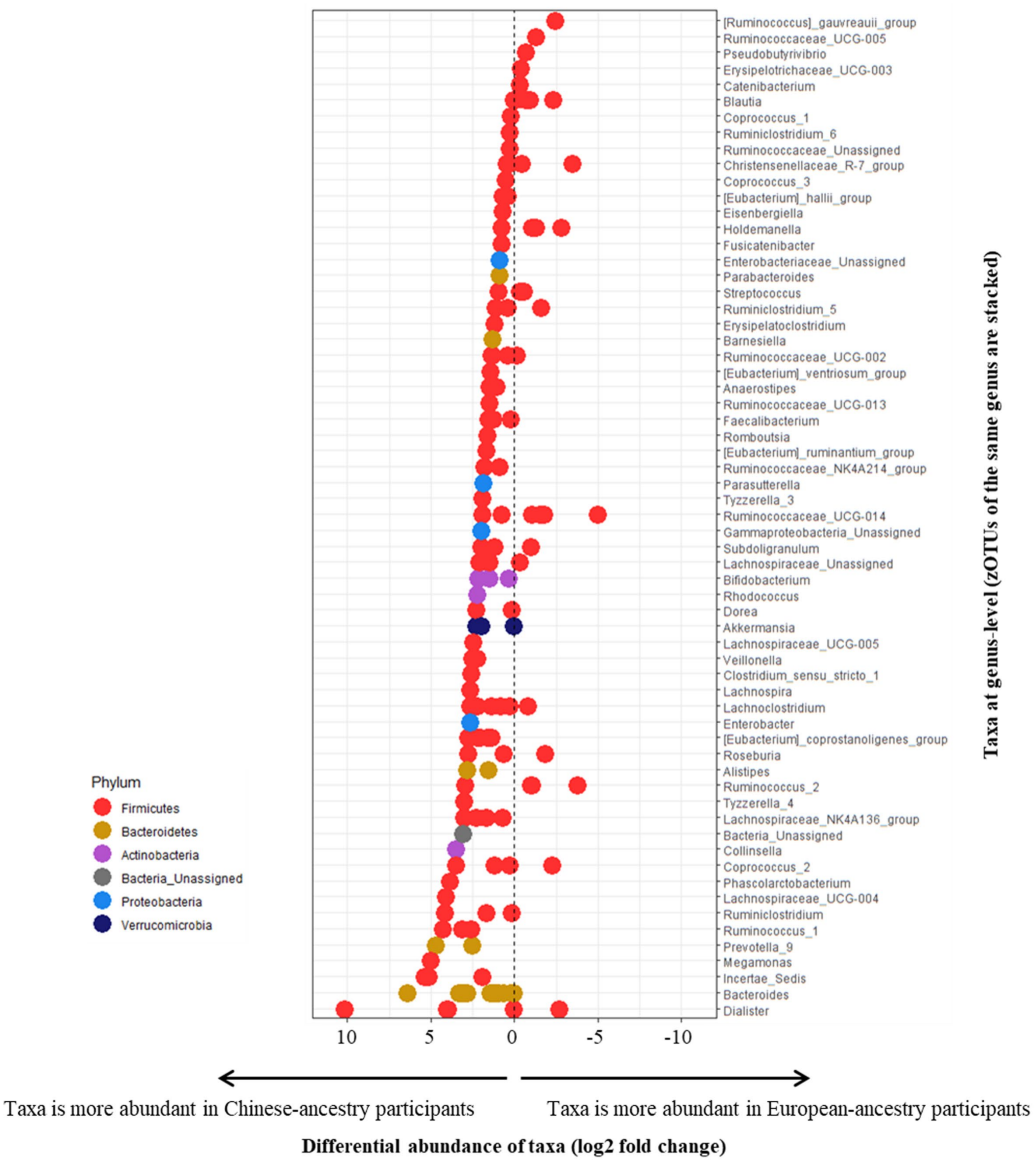
Differential abundance of zOTUs (based on European-ancestry participants as the reference factor, representing an abundance of 0). All positively measuring zOTUs represent taxa more abundant in Chinese-ancestry participants, and negatively measuring zOTUs represent taxa more abundant in European-ancestry participants. zOTUs measuring 0 indicate a balanced distribution of taxa across both cohorts. All zOTUs from the same genus are stacked horizontally (based on their respective differential abundance). zOTUs are color-coded by their respective phyla.

Differential abundances of zOTUs across the cohorts were explored using European-ancestry participants as the reference factor (representing an abundance of 0), with the majority of differentially abundant zOTUs being more abundant among the Chinese-ancestry participants (Figure 4). No zOTUs from phyla other than *Firmicutes* were over-represented among European-ancestry individuals, but different members of the same genus were often observed to be differentially enriched across either ethnic cohort.

## 4. Discussion

The underlying hypothesis for this study was that two ethnically distinct communities, both in the same country of residence and with increased risk of T2D-onset based on fasting dysglycaemia, would differ in their gut (fecal) microbiota profiles. To address this point, we conducted a direct comparison of fecal-derived 16S rRNA gene sequence data from two separate clinical trials investigating prediabetes in Chinese-ancestry and European-ancestry communities living in New Zealand. The two cohorts were matched for mean FPG during selection of participants for this sub-analysis. Coincidentally, Chinese-ancestry participants were enrolled at a lower BMI than European-ancestry participants, likely due to their higher propensity for T2D onset at lower BMI as is consistent with the aforementioned TOFI profile. Microbiota analyses were performed separately for samples from each clinical trial, but in the same laboratory at the University of Auckland, and the methodologies utilized for fecal sample collection and processing (DNA extraction, PCR and sequencing) were identical. While sample type for FPG measurement differed between the cohorts, both are internationally accredited methods and all participants included in the current analysis were identified with prediabetes.

Although not directly assessing the TOFI phenotype, which requires measurement of ectopic fat infiltration, our findings show this Asian cohort with prediabetes to be characterized by similar age and FPG but significantly lower body weight, BMI, total and abdominal body fat, and higher hyperinsulinaemia compared to the European-ancestry cohort. This aligns with the hypothesis that Chinese-ancestry participants may have increased T2D susceptibility at lower BMI with accompanying lower total and upper body (abdominal) fat. In a previous larger study using MRI techniques to assess body composition in a different Chinese-ancestry cohort also residing in Auckland, we have shown % pancreas and visceral fat to have the strongest positive correlation with FPG, independent of age and % total body fat (Sequeira et al., 2022). In the context of gut microbiota structure, we observed significant differences between the two ethnic cohorts, with key differences in relative abundances of the *Firmicutes*, *Bacteroidetes*, and *Actinobacteria* bacterial phyla. While our microbiota findings could in principle reflect the substantial difference in, for example, BMI between the two cohorts, we note that ethnicity but not BMI was statistically significant in our analyses. The greater relative abundance of *Firmicutes* observed in the European-ancestry cohort may have contributed to this cohort’s lower overall Shannon diversity. These findings are consistent with those from recent large-scale studies in the USA (Brooks et al., 2018) and Netherlands (Deschasaux et al., 2018), in ethnically diverse participants from common urban environments. Here in our current analysis, as per PERMANOVA analyses, ethnicity also had a strong influence on gut bacterial composition in participants with fasting dysglycaemia consistent with prediabetes. It is unlikely that genetic factors are the only important characteristics that may be driving microbiota diversity, but we propose that they are likely to contribute. Interestingly, there is some evidence that such differences mitigate over time as immigrants adapt to a Western lifestyle (Vangay et al., 2018), however this could not be assessed in our current cohorts as data on years of residence in New Zealand were not collected in the original studies. Conversely, no significant effect of ethnicity on gut microbiota composition was observed in a large Middle Eastern cohort (Rothschild et al., 2018), though this could potentially be due to more homogenous local dietary and lifestyle factors in comparison to the social diversity observed in Western countries.

In a recent multi-omics study, Ang et al. (2021) investigated the gut microbiome profiles of 46 East Asian and White individuals resident in the San Francisco Bay area, across a range of body composition profiles from lean to those with obesity, and showed a strong ethnically-associated distinction between the two cohorts. Consistent with our findings, they reported a high proportion of *Firmicutes* in individuals with obesity and also noted the East Asian cohort to have a higher relative abundance of *Bacteroidetes*. Unexpectedly, although they were able to explain some of the observed gut microbiota differences by ethnicity and geography, they did not identify any associations with host diet. Methodological differences in dietary data collection between the two origin studies in our current analysis prevented a quantitative comparison of background diet, but this would be of interest to determine. Moreover, the observed ethnicity-associated differences in the study of Ang and colleagues were stronger in lean individuals, indicating a potential capacity for obesity-related influences to “overwrite” or diminish ethnicity-associated microbial signatures. Although they suggest the possibility of a shared ethnicity-independent microbiome pattern that increases host susceptibility to obesity onset (Ang et al., 2021), such outcomes elevate the importance of controlling for ethnicity (in addition to demographic and metabolic parameters) in studies exploring associations between the gut microbiota and diseases of interest.

All samples analyzed in this study were obtained from participants with overweight or obesity and prediabetes. Perhaps unsurprisingly, among the highly abundant zOTUs (≥1% overall 16S rRNA gene relative sequence abundance) observed, several bacterial genera were previously associated with prediabetes as well as frank T2D. Bacteria negatively correlated with diabetes such as *Faecalibacterium* (4 zOTUs) (Furet et al., 2010; Graessler et al., 2013; Karlsson et al., 2013; Zhang et al., 2013; Remely et al., 2014; Murphy et al., 2017; Gao et al., 2018; Salamon et al., 2018; Ghaemi et al., 2020; Molinaro et al., 2020), *Bifidobacterium* (Wu et al., 2010, 2017; Xu et al., 2015; Candela et al., 2016; Pedersen et al., 2016; Sedighi et al., 2017; Barengolts et al., 2018; Gao et al., 2018; Ghaemi et al., 2020) (2 zOTUs in Chinese-ancestry participants) and *Bacteroides* (Munukka et al., 2012; Zhang et al., 2013, 2020; Candela et al., 2016; Yamaguchi et al., 2016; Lippert et al., 2017; Ghaemi et al., 2020) were found in both cohorts, with the further addition of *Akkermansia* (Everard et al., 2013; Zhang et al., 2013, 2020; Greer et al., 2016; Allin et al., 2018; Ghaemi et al., 2020) in Chinese-ancestry participants. Both cohorts also harbored two bacteria previously shown to be positively associated with T2D, *Blautia* (Zhang et al., 2013, 2020; Egshatyan et al., 2016; Inoue et al., 2017; Lippert et al., 2017; He et al., 2018) (3 zOTUs in European-ancestry participants) and *Ruminococcus* (Zhang et al., 2013; Candela et al., 2016; Patrone et al., 2016; Allin et al., 2018; Salamon et al., 2018; Molinaro et al., 2020) (2 zOTUs in European-ancestry participants and 3 zOTUs in Chinese-ancestry participants). While butyrate-producing bacteria are generally considered to be negatively associated with T2D co-morbidities (Vital et al., 2017) such as adipocyte inflammation (Wang et al., 2015) and insulin resistance (Gao et al., 2009, 2019), butyrate producers in the genera *Pseudobutyrivibrio* (Xu et al., 2015; Nie et al., 2019; Zhao et al., 2019), *Coprococcus* (Chávez-Carbajal et al., 2019) and *Anaerostipes* (Org et al., 2017; Zhao et al., 2019) have previously been linked with T2D and metabolic syndrome, hence their precise role remains unclear. In this study, both cohorts contained a high abundance of *Pseudobutyrivibrio* and *Coprococcus*, as well as *Anaerostipes* in the Asian cohort. Nevertheless, and as evident from our differential abundance analyses (Figure 4), there can exist considerable variation at the zOTU level within a given genus (such as *Dialister, Ruminococcaceae_UCG-014*), with different members often differentially enriched in either ethnic cohort despite their close genetic distances.

## 5. Conclusion

Our findings have revealed a clear distinction between the gut microbiota profile of two disparate communities from the same country of residence, predominantly from the city of Auckland. These were a European**-**ancestry cohort with prediabetes and a Chinese**-**ancestry cohort with prediabetes plus phenotype traits consistent with TOFI (i.e., dysglycaemia and possible early pancreatic β-cell dysfunction despite a lean external appearance). While sex distribution did differ between the two cohorts, our findings support the notion that ethnicity, defined as self-perceived cultural affiliation encompassing diet, lifestyle and genetic components, may at least partially explain some of the commonly observed high variation in the gut microbiota among individuals. However, even after accounting for ethnicity we observed substantial inter-individual variation in zOTU-level assignments within each cohort. Human diet, lifestyle and genetics are broad characteristics that have been proposed to shape gut microbiota composition, and are likely all contributing in our current study together with other factors that may affect these highly host-specific variables. A notable recent gut microbiome study conveyed the importance of controlling for frequency of alcohol consumption and bowel movement quality (based on the Bristol stool scale), among other factors (Vujkovic-Cvijin et al., 2020). Social factors such as family size, living conditions, income, religion, education and employment in turn may all have an impact on diet and lifestyle and therefore should also be considered. Our findings underscore the potential influence of ethnicity on gut microbiota composition in the context of individuals with overweight and prediabetes.

## Data availability statement

The datasets presented in this study can be found in online repositories. The names of the repository/repositories and accession number(s) can be found at: https://www.ncbi.nlm.nih.gov/genbank/, SRA Bioproject PRJNA900794 for the Chinese-ancestry cohort. The data for the European-ancestry cohort is held in the European Nucleotide Archive under accession number PRJEB43667.

## Ethics statement

The studies involving humans were approved by Health and Disabilities Ethics Committee (HDEC), New Zealand. The studies were conducted in accordance with the local legislation and institutional requirements. The participants provided their written informed consent to participate in this study.

## Author contributions

AM: metadata collection and database entry, performed lab work (DNA extraction, amplification, purification), processed sequence data, statistical analyses, data interpretation, and primary manuscript preparation. LL: conducted the Tū Ora clinical trial. IS-B: study design and clinical trial oversight. MS: conducted the PREVIEW clinical trial. MH: performed lab work (DNA extraction, amplification, purification). DB: statistical analyses. MF: PREVIEW clinical trial PI and study design. AR: PREVIEW project and clinical trial fund raiser, study design. SP: fund raiser, study design, trial supervision, data interpretation, and manuscript preparation. MT: fund raiser, data interpretation, and manuscript preparation. All authors reviewed and edited the manuscript.
