# Supplementary material for: Gut microbiota profiles in two New Zealand cohorts with overweight and prediabetes: a Tū Ora/PREVIEW comparative study
Source: Front Microbiol. 2023 Nov 16;14:1244179. doi: 10.3389/fmicb.2023.1244179 (PMC10687470; doi:10.3389/fmicb.2023.1244179)
Supplement: Supplementary file 1 [file Data_Sheet_1.PDF]

Supplementary Table 1: Complete output from permutational multivariate analysis of variance (PERMANOVA) of 16S rRNA gene-based gut microbiota data, testing for associations between ethnicity and key demographic variables (i.e. age and sex) and metabolic variables (i.e. BMI and FPG).

	**Df**	**SumsOfSqs**	**MeanSqs**	**F.Model**	**R2**	**Pr(>F)**	
Ethnicity	1	1.036	1.036	3.477	0.051	<0.001	*
Age	1	0.272	0.272	0.911	0.013	0.584	
Sex	1	0.249	0.249	0.836	0.012	0.689	
BMI	1	0.149	0.149	0.501	0.007	0.990	
FPG	1	0.234	0.234	0.785	0.011	0.771	
Ethnicity:Age	1	0.255	0.255	0.855	0.012	0.662	
Ethnicity:Sex	1	0.252	0.252	0.845	0.012	0.680	
Age:Sex	1	0.284	0.284	0.954	0.014	0.513	
Ethnicity:BMI	1	0.313	0.313	1.049	0.015	0.373	
Age:BMI	1	0.193	0.193	0.646	0.009	0.921	
Sex:BMI	1	0.259	0.259	0.868	0.013	0.651	
Ethnicity:FPG	1	0.369	0.369	1.239	0.018	0.183	
Age:FPG	1	0.387	0.387	1.299	0.019	0.142	
Sex:FPG	1	0.235	0.235	0.789	0.011	0.767	
BMI:FPG	1	0.319	0.319	1.069	0.016	0.356	
Ethnicity:Age:Sex	1	0.143	0.142	0.478	0.007	0.993	
Ethnicity:Age:BMI	1	0.432	0.432	1.451	0.021	0.074	
Ethnicity:Sex:BMI	1	0.179	0.179	0.599	0.009	0.954	
Age:Sex:BMI	1	0.168	0.168	0.563	0.008	0.968	
Ethnicity:Age:FPG	1	0.327	0.327	1.096	0.016	0.323	
Ethnicity:Sex:FPG	1	0.249	0.249	0.835	0.012	0.701	
Age:Sex:FPG	1	0.225	0.225	0.755	0.011	0.807	
Ethnicity:BMI:FPG	1	0.217	0.217	0.728	0.011	0.845	
Age:BMI:FPG	1	0.301	0.301	1.009	0.015	0.425	
Sex:BMI:FPG	1	0.423	0.423	1.419	0.021	0.079	
Ethnicity:Age:Sex:BMI	1	0.226	0.226	0.760	0.011	0.808	
Ethnicity:Age:Sex:FPG	1	0.150	0.150	0.504	0.007	0.987	
Ethnicity:Age:BMI:FPG	1	0.335	0.335	1.124	0.016	0.294	
Ethnicity:Sex:BMI:FPG	1	0.186	0.185	0.622	0.009	0.941	
Age:Sex:BMI:FPG	1	0.159	0.159	0.532	0.008	0.978	
Ethnicity:Age:Sex:BMI:FPG	1	0.327	0.327	1.095	0.016	0.328	
Residuals	39	11.625	0.298		0.568		
Total	70	20.476			1.000		

The statistical environment R was used to conduct analyses, with a p-value <0.05 considered to be statistically significant. Statistical analysis was performed using all available data from all participants. Significance of variance was determined using the R package 'vegan', with PERMANOVA performed using the 'adonis' function, performing 9999 unrestricted permutations of the raw data. The following code was used to perform this analysis:

```
library(vegan)
OTU_table <- read.csv("OTU_table.csv")
Metadata <- read.csv("Metadata.csv")
OTU_data <- t(OTU_table[,-1]) #Transposing otu data to ensure it matches the data
structure of metadata (ie. Sample names are oriented in either columns or rows across
both corresponding files)
adonis(OTU_data ~ Ethnicity * Age * Sex * BMI * FPG, data = Metadata, permutations =
9999)
```
